# SIRT7 and p53 interaction in embryonic development and tumorigenesis

**DOI:** 10.3389/fcell.2023.1281730

**Published:** 2024-01-03

**Authors:** Berta N. Vazquez, Irene Fernández-Duran, Yurdiana Hernandez, Shahriar Tarighi, Joshua K. Thackray, Maria Espinosa-Alcantud, Poonam Kumari, Alessandro Ianni, Lionel Cesaire, Thomas Braun, Manel Esteller, Jay Tischfield, Alejandro Vaquero, Lourdes Serrano

**Affiliations:** ^1^ Chromatin Biology Laboratory, Josep Carreras Leukaemia Research Institute (IJC), Badalona, Spain; ^2^ Unitat de Citologia i Histologia, Departament de Biologia Cel.lular, de Fisiologia i d’Immunologia, Universitat Autònoma de Barcelona (UAB), Cerdanyola del Vallès, Barcelona, Spain; ^3^ Department of Genetics, Human Genetics Institute of New Jersey (HGINJ), Rutgers University, Piscataway, NJ, United States; ^4^ Department of Cardiac Development and Remodeling, Max-Planck-Institute for Heart and Lung Research, Bad Nauheim, Germany; ^5^ Department of Science, Borough of Manhattan Community College (BMCC), The City University of New York (CUNY), New York, NY, United States; ^6^ Cancer Epigenetics Group, Josep Carreras Leukaemia Research Institute (IJC), Barcelona, Spain; ^7^ Centro de Investigacion Biomedica en Red Cancer (CIBERONC), Madrid, Spain; ^8^ Institucio Catalana de Recerca I Estudis Avançats (ICREA), Barcelona, Catalonia, Spain; ^9^ Physiological Sciences Department, School of Medicine and Health Sciences, University of Barcelona (UB), Barcelona, Catalonia, Spain

**Keywords:** p53, SIRTUIN, Sirt7, embryonic development, tumor suppressor, gene expression, epithelial to mesenchymal transition

## Abstract

p53 is a hallmark tumor suppressor due in part to its role in cell cycle progression, DNA damage repair, and cellular apoptosis; its protein activity interrelates with the Sirtuin family of proteins, major regulators of the cellular response to metabolic, oxidative, and genotoxic stress. In the recent years, mammalian Sirtuin 7 (SIRT7) has emerged as a pivotal regulator of p53, fine-tuning its activity in a context dependent manner. SIRT7 is frequently overexpressed in human cancer, yet its precise role in tumorigenesis and whether it involves p53 regulation is insufficiently understood. Depletion of SIRT7 in mice results in impaired embryo development and premature aging. While p53 activity has been suggested to contribute to tissue specific dysfunction in adult *Sirt7* ^*−/−*^ mice, whether this also applies during development is currently unknown. By generating SIRT7 and p53 double-knockout mice, here we show that the demise of SIRT7-deficient embryos is not the result of p53 activity. Notably, although SIRT7 is commonly considered an oncogene, SIRT7 haploinsufficiency increases tumorigenesis in p53 knockout mice. Remarkably, in specific human tumors harboring p53 mutation, we identified that SIRT7 low expression correlates with poor patient prognosis. Transcriptomic analysis unveils a previously unrecognized interplay between SIRT7 and p53 in epithelial-to-mesenchymal transition (EMT) and extracellular matrix regulation with major implications for our understanding of embryonic development and tumor progression.

## Introduction

The TP53 gene encodes the transcription factor p53, known for its crucial roles in tumor suppression (Aubrey et al., 2016). Posttranslational modifications, such as lysine acetylation leads to its stabilization and accumulation (Hafner et al., 2019). p53 serves as a vital component in genome integrity surveillance. In the presence of genotoxic stress, p53 triggers cell cycle arrest, facilitates DNA damage repair, and, if necessary, orchestrates cellular apoptosis and senescence (Williams and Schumacher, 2016). Deficiencies in DNA repair factors in mice result in early p53 hyperactivation and embryo death. Consequently, p53 gene deletion rescues the embryonic lethality observed in some of these mouse models (Williams and Schumacher, 2016). p53 loss of function is a common occurrence in cancers and often correlates with an unfavorable prognosis, and the germline deletion of p53 in both humans and mice predisposes individuals to an increased cancer incidence (Bieging et al., 2014; Mantovani et al., 2019).

The Sir2 family of proteins, known as Sirtuins or SIRTs, are protein deacetylases and ADP-ribosyltransferases that require the cofactor NAD^+^ for their activity. They play essential roles in genome maintenance, metabolism, and cellular stress responses (Bosch-Presegue and Vaquero, 2014). In mammals, there are seven Sirtuin members, namely, SIRT1-SIRT7, which exhibit distinct cellular localizations. While Sirtuins are widely recognized for their involvement in health-span pathways and longevity (Imai and Guarente, 2016; Vazquez et al., 2020), they also play central roles in cancer pathogenesis, acting as context-specific oncogenes or tumor suppressors (Bosch-Presegue and Vaquero, 2011). Mammalian SIRT7, a nuclear Sirtuin, is clearly significant in maintaining genome integrity (Vazquez et al., 2016; Vazquez et al., 2019a; Vazquez et al., 2019b). It is critically involved in repairing DNA double-strand breaks (DSBs), which are highly cytotoxic and mutagenic lesions, by modulating non-homologous end joining (NHEJ) and homologous recombination (HR) repair pathways (Li et al., 2016; Vazquez et al., 2016). Additionally, SIRT7 plays a role in silencing LINE-1 retrotransposons, which are known to induce DSBs and cellular toxicity when upregulated. Notably, SIRT7 dynamically regulates the function of the p53 protein through both direct and indirect mechanisms. In myocardiocytes, SIRT7 interacts with and deacetylates p53, suppressing its pro-apoptotic activities with major implications in the molecular mechanisms underlying myocardial pathologies (Vakhrusheva et al., 2008; Sun et al., 2018). Conversely, in cellular models of UV light-induced genotoxic stress (Ianni et al., 2021) and glucose deprivation (Lu et al., 2020), SIRT7 stabilizes p53 protein levels by disrupting the interaction between p53 and the E3 ubiquitin ligase MDM2, thereby inhibiting p53 proteasomal degradation. These studies collectively underscore the cell type and stress-dependent fine-tuning of p53 activity by SIRT7 and reinforce the critical role of SIRT7 in the overall genome surveillance process.

Deficiency of SIRT7 in mice leads to a premature aging syndrome that affects both somatic (Vazquez et al., 2016) and reproductive tissues (Vazquez et al., 2019b; Vazquez et al., 2020). Moreover, *Sirt7* ^*−/−*^ mice are born at submendelian ratios, indicating disrupted embryonic development (Vazquez et al., 2017). The underlying molecular cause of this embryonic lethality remains unanswered. Considering the association between SIRT7, DSB DNA damage repair, and p53 regulation (Vakhrusheva et al., 2008), we hypothesize that p53 activation may contribute to the death of *Sirt7* ^*−/−*^ embryos and that deleting the p53 gene could alleviate this phenotype, albeit with increased genome instability and cancer risk in adulthood. However, our findings demonstrate that the simultaneous depletion of SIRT7 and p53 has a detrimental effect on embryo development. Specifically, *Sirt7* ^*−/−*^ *p53* ^*−/−*^ mice are non-viable, and *SirT7* ^*+/−*^ *p53* ^*−/−*^ mice exhibit increased embryo lethality. Furthermore, surviving *Sirt7* ^*+/−*^ *p53* ^*−/−*^ mice display increased tumorigenesis compared to *Sirt7* ^*+/+*^ *p53* ^*−/−*^ mice, which correlates with a metastatic outcome of cancer progression. In line with the findings observed in mice, *in silico* analysis reveals a distinct subset of human tumors, in which low levels of SIRT7 expression are associated with reduced patient survival in a p53-dependent manner. Transcriptomic analysis on primary mouse embryonic fibroblasts (MEFs) and tumor datasets reveals that both p53 and SIRT7 co-regulate gene expression programs associated with EMT and the extracellular matrix compartment. EMT is a critical pathway involved in normal tissue morphogenesis and organogenesis, but it is commonly dysregulated in cancer, particularly during metastasis (Campbell, 2018). These findings shed light on a previously unrecognized interplay between SIRT7 and p53, specifically in EMT pathways regulation. This interplay holds significant implications for embryogenesis and cancer progression.

## Material and methods

### Mice

129Sv mice *Sirt7* ^*−/−*^ were originally purchased from the Jackson Laboratories. Mixed 129sv/BL6 *p53* ^*−/−*^ mice were kindly provided by Dr. Bunting (Rutgers University, United States). After crossing *Sirt7* ^*+/−*^ and *p53* ^*+/−*^ mice, crossing *Sirt7* ^*+/−*^ *p53* ^+/−^ double heterozygous mice was used as a mating strategy. During the euthanasia process, animals were subjected to CO_2_ gas exposure at a flow rate carefully calibrated to achieve a displacement of 30%–70% of the chamber volume per minute. Ensuring humane conditions, each animal was provided enough space to stand on all four feet and had sufficient room to turn around within the chamber. Animal studies were conducted in accordance with Rutgers University IACUC policies.

### Cell culture, infections, and transfections

WT and *Sirt7* ^*−/−*^ mouse embryonic fibroblasts (MEFs) were obtained as previously described in (Serrano et al., 2013). Embryonic stem cells (ESCs) and embryo body (EB) differentiation was conducted as described elsewhere (Tian et al., 2019). For p53 and SIRT7 knockdown, retroviral (for MEFs) or lentiviral (for ESCs) particles were first produced in 293FT cells. Briefly, pSUPER retro puro scramble shRNA and pSUPER retro puro p53 shRNA retroviral plasmids were transfected using polyethylenimine into 293FT cells. Two days later, viral supernatant was collected and passed through a 0.45 μm syringe filter. The viral supernatant was complemented with polybrene to a final concentration of 4 μg/mL and MEFs were treated with fresh viral supernatant every 3 h. When three rounds of infection were completed, viral supernatant was replaced with fresh media. Two days after infection, selection with puromycin (1 μg/mL) was initiated and continued until no alive cells were observed in control cells infected with a non-containing puromycin selection marker vector.

### Colony formation

250 cells from each genotype were plated in triplicate in 6 well plates, cultured for 8 days, and fixed with 0.5% glutaraldehyde for 20 min at room temperature, followed by staining with 0.02% crystal violet 3 h at room temperature. Wells were washed three times with dH_2_O and dried overnight. After imaging the plates, cellular mass was quantified by extracting cell-bound crystal violet in 10% acetic acid at room temperature. Once all wells were completely distained, equal amounts were transferred to a 96-well plate in triplicate and absorbance was read at 595 nm. Cell content was relatively quantified using the average absorbance of WT lines. Experiments were conducted in three independent WT and *Sirt7* ^*−/−*^ MEFs lines.

### Wound-healing assay

Cells were plated at 90% confluence in 6-well plates in triplicates. The following day, a straight scratch wound was established with a 1 mL sterile pipette tip across the confluent monolayer. One PBS 1X wash was performed to remove cell debris and pictures of the scratch at time h = 0 was taken using a brightfield microscope. Wound closure was monitored by taking pictures of the same region at 9h, 24h and 30h. Wound areas were measured using FIJI ImageJ and the relative recovered area was calculated. Experiments were conducted in three independent WT and *Sirt7* ^*−/−*^ MEFs lines.

### Apoptosis assay

For cell survival analysis, MEFs and cell lines were stained with Annexin V-FITC (eBiosciences) and 7AAD (Pharmingen) using standard methods. FACS analysis was performed using FACSCanto II (BD Biosciences) and FlowJo 7/8 (Tree Star).

### Histopathology

Gross anatomy descriptions of sacrificed animals were conducted in collaboration with Animal Services at Rutgers University. Animals were regularly monitored for tumor development, particularly in the *p53* ^*−/−*^ mice. Mice with visible masses underwent a thorough external examination to assess general health, behavior, movement, skin condition, and other abnormalities. When signs of sickness were identified, humane euthanasia was carried out as an endpoint, followed by necropsy procedures adhering to AVMA guidelines and Rutgers University protocols. Proper personal protective equipment (PPE) was utilized during the necropsy, ensuring aseptic conditions. During the necropsy, the animal’s weight was recorded, and all external openings were examined (ears, eyes, nose, anus, genital openings, and oral cavity). A skin incision was made along the belly to expose the abdominal organs, glands, and lymph nodes. Additionally, lateral, and transverse cuts were made to examine the organs within the chest cavity. When tumors were identified, the macroscopic features of each tumor were carefully documented, including location, size, texture, adherence, and signs of visible metastasis. When submitting for histopathology, mice were subsequently fixed with formalin and processed by Charles River Research Animal Diagnostic Services. A clinical history for each animal was provided, including date of birth and death and gross anatomy findings. Histopathology analysis included gross examination of fixed animals, extended organ survey, and organ H&E slide preparation and interpretation. The pathology report included gross and microscopic observations and diagnosis regarding tumor type, localization and range of malignancy (metastasis).

### Patient survival analysis

The cBioPortal for Cancer Genomics (http://www.cbioportal.org) (Cerami et al., 2012; Gao et al., 2013) was used to interrogate the interplay of SIRT7 and p53 in human samples, specifically the TCGA Firehose Legacy datasets, which contains pathological data, a description of germline mutations, and mRNA expression data. The analysis involved categorizing the datasets based on p53 germline mutations and further subdividing them according to SIRT7 mRNA expression z-scores derived from all samples. To create distinct SIRT7^HIGH^ and SIRT7^LOW^ groups, specific expression thresholds were determined based on the minimum increase and reduction necessary to observe a significant impact on overall patient survival. For BIC, SA and BLGG, SIRT7^HIGH^ and SIRT7^LOW^ z-scores are ≤+ 1 and ≥-1 respectively, for HNSCC, ≤+0.75 and ≥-0.75, for sarcoma and OSC, ≤+0.5 and ≥-0.5, and for BUC ≥ −0.25, ≤+0.25. Kaplan-Meier survival curves were subsequently generated. Log-rank test (Mantel-Cox test) was performed automatically by the cBioPortal software, and a *p*-value <0.05 considered to indicate a statistically significant difference.

### RNA-seq analysis

We obtained from the Gene Expression Omnibus two previously published RNA-seq datasets for *Sirt7* ^−/−^ or *p53* ^−/−^ and their respective controls (GSE128893 and GSE46240, respectively). Data was analyzed as previously described (Vazquez et al., 2019a; Simonet et al., 2020). For each dataset, we extracted raw reads fastq from SRA archives and trimmed raw reads with trimmomatic (v0.36) (Bolger et al., 2014) and read quality was verified using fastqc (v0.11.5) on reads prior and post trimming. Trimmed reads were submitted for alignment (Kim et al., 2019) against the GRCm38 reference genome using parameters “—dta--no-softclip” and results written to SAM files, which were subsequently converted to BAM format, sorted, and indexed with samtools (v0.1.9) (Li et al., 2009). Transcript alignment counts we quantified using StringTie (v1.3.3) (Pertea et al., 2016) against the mouse transcript annotations from GRCm38 reference GTF with parameters “–e.” Data was converted from Ballgown format to count matrix format using the script “prepDE.py” supplied by StringTie authors. Count matrices were imported into DESeq2 and differential gene expression analysis performed (Love et al., 2014). Both experiments consisted of a two-variable design: WT or mutant cells which were either left untreated or were treated in some manner (glucose starvation in the case of SIRT7, dox in p53). In order to benefit from better dispersion estimates, we opted to include all samples for each experiment, and use the DESeq2 model specified as (∼genotype + treatment + genotype:treatment). The DESeq2 model was fit and log2 fold changes shrunk with apeglm (Zhu et al., 2019), and only the main effect of the genotype factor was considered. Significant differentially expressed genes were considered as genes with adjusted *p*-value <0.05 and a |Log2 fold change| > 0.25. Results reported indicate the log2 fold change of gene expression in each mutant relative to their respective controls (Xie et al., 2021).Venn diagrams were generated by taking the list of differentially expressed gene symbols in *Sirt7* ^*−/−*^ vs. WT and *p53* ^*−/−*^ vs. WT, and uploading them to a freely available venn diagram web application (http://bioinformatics.psb.ugent.be/webtools/Venn/). Genes which were significantly upregulated in both *Sirt7* ^*−/−*^ and *p53* ^*−/−*^ cells, relative to their respective controls, were submitted to Gene Ontology analysis using Enrichr. Heatmaps showing changes in gene expression were generated using log2 fold changes calculated by DESeq2 and plotted using seaborn.

### RNA isolation and quantitative PCR

According to the manufacturer’s instructions, total RNA from cell lines was isolated using the Promega Maxwell RNA purification kit and from tissue samples using RecoverAll™ Multi-Sample RNA/DNA Workflow (Invitrogen). cDNA was generated using the Transcriptor First Strand cDNA Synthesis Kit (Roche Life Science). Quantitative PCR was performed with SYBR Green and the QuantStudio quantitave PCR system (Life Technologies), and the target genes’ relative expressions were normalized to Actin mRNA levels. Primer sequences are as follows: mAdamtS5-F 5′-CTG​CCT​TCA​AGG​CAA​ATG​TGT​GG-3′ and mAdamtS5-R 5′-CAA​TGG​CGG​TAG​GCA​AAC​TGC​A-3’; mREM1-F 5′-GCT​TCT​TGG​AGA​CCC​TGG​TGT​T-3′ and mREM1-R 5′-TCC​ACT​GAC​AGC​GTT​CTC​TCG​T-3′, Adam33-F 5′-CTT​GGA​AGC​AGG​AGA​AGA​GTG​C-3′ and Adam33-R 5′-CAG​CAA​TCA​CCG​TGG​GCA​CAT​T-3’; mTGFβ2-F 5′- TTG​TTG​CCC​TCC​TAC​AGA​CTG​G-3′ and mTGFβ2-R 5′-GTA​AAG​AGG​GCG​AAG​GCA​GCA​A-3’; β2microglobulin-F 5′AGA​CTG​ATA​CAT​ACG​CCT​GCA-3’; β2microglobulin-R 5′-GCA​GGT​TCA​AAT​GAA​TCT​TCA-3’; mACTBF 5′ TGA​CCC​TGA​AGT​ACC​CCA​TTG 3’; mACTBR 5′ CCA​TGT​CGT​CCC​AGT​TGG​TAA 3’; mSIRT7F 5′ TCT​CAG​AGC​TCC​ATG​GGA​AT 3’; mSIRT7R; 5′ GAA​GGG​CAG​TAC​GCT​CAG​TC 3’.

## Results

### Loss of p53 exacerbates embryonic lethality of SIRT7 deficient mice

**FIGURE 1:**
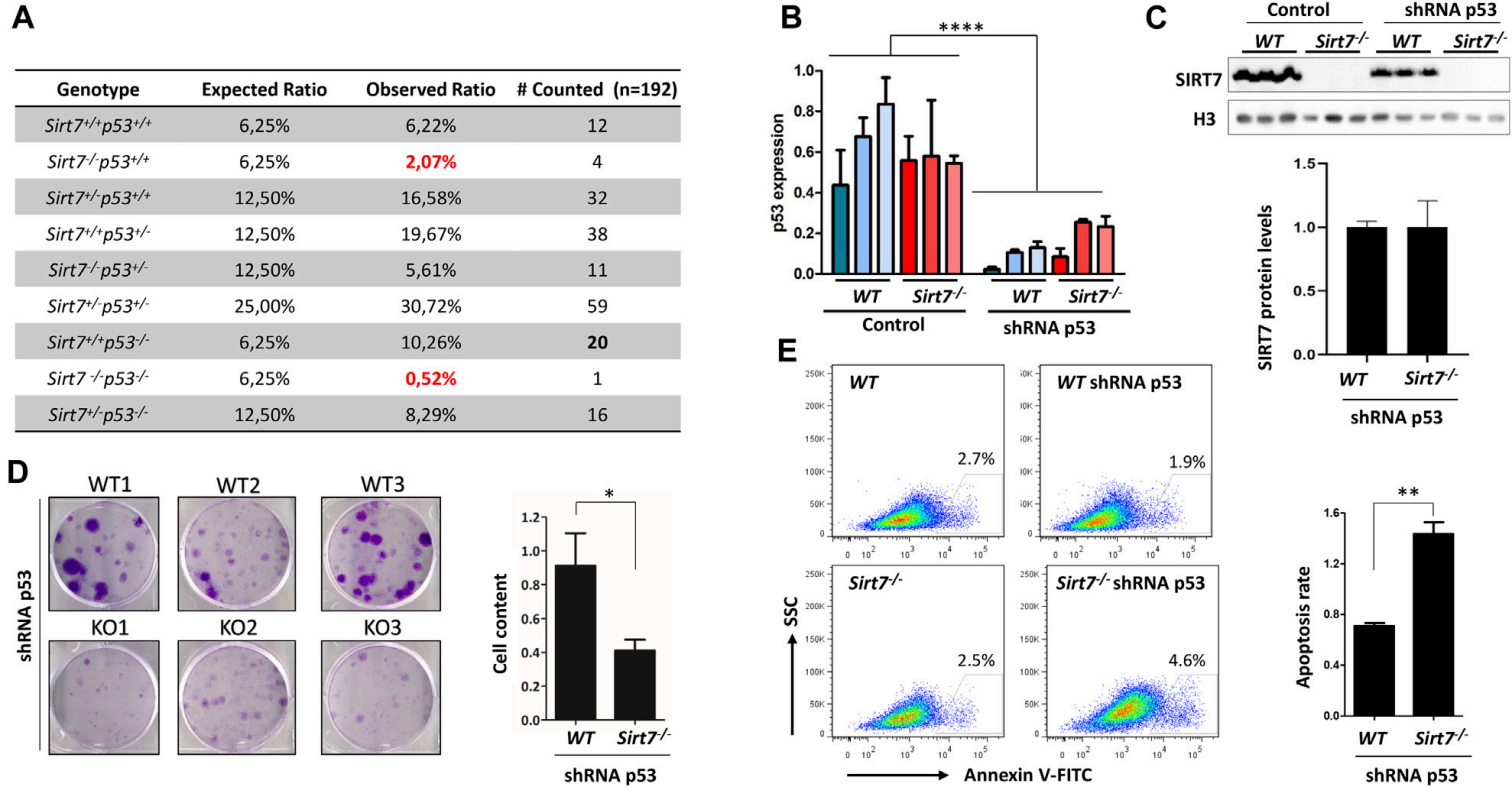
SIRT7 and p53 concomitant depletion in embryonic cells impair embryo development, cell growth, and survival. **(A)** Homozygous p53 deletion does not rescue embryonic lethality of *Sirt7* ^*−/−*^ mice. Mendelian ratios from *SirT7* ^*+/−*^ *p53* ^*+/−*^ mouse crosses (*n* = 192 pups; *p*-value <0.001 by χ2). **(B,C)** WT and *Sirt7* ^*−/−*^ MEFs were infected with retroviral vectors expressing an shRNA control or targeting p53. Levels of SIRT7 and p53 were analyzed by RT-PCR and by Western blot. **(D)** Colony formation assays in WT and *Sirt7* ^*−/−*^ MEFs expressing an shRNA targeting p53. Quantification represents relative crystal violet staining absorbance (Abs 550 nm). **(E)** Cell apoptotic assays in same cell lines as in **(D)**, using Annexin V staining. Apoptosis rate was obtained by diving apoptotic values of shRNA p53 expressing MEFs by the apoptotic values of parental non-transformed cells. **(B–D)** Otherwise indicated, quantification represents the mean ± SEM of three independent experiments conducted in three independent cell lines. Data information: *p < 0.05; **p < 0.01; ***p < 0.001; ****p < 0.0001 by two-way ANOVA **(B)** and *t*-test **(C–E)**.

We previously reported that SIRT7 depletion in mice leads to partial embryonic lethality as *Sirt7* ^*−/−*^ mice are born at submendelian ratios from *Sirt7* ^*+/−*^ heterozygous crosses (Vazquez et al., 2016). In addition, *Sirt7* ^*−/−*^ primary MEFs have impaired DNA DSB repair and genome instability (Vazquez et al., 2017). Because there is a well stablished link between unrepaired DNA DSBs and p53-dependent apoptosis in embryos (Williams and Schumacher, 2016), we aimed to determine whether p53 deletion would rescue the embryonic lethality of SIRT7 deficient mice by generating and analyzing the progeny of *Sirt7* ^*+/−*^and *p53* ^*+/−*^ mouse crosses (Figure 1A). Consistent with our previous observations (Vazquez et al., 2016), the *Sirt7* ^*−/−*^ *p53* ^*+/+*^ genotype was underrepresented in the mice offspring with a 4-fold reduction from the expected Mendelian ratios while *Sirt7* ^*+/+*^ *p53* ^*−/−*^ mice were born following expected rates with no apparent defects, also in agreement with previous reports (Donehower et al., 1992). Strikingly, from 192 genotyped pups, we only detected one *Sirt7* ^*−/−*^ *p53* ^*−/−*^ mouse and a significant reduction of *Sirt7* ^*+/−*^ *p53* ^*−/−*^ mice compared with *Sirt7* ^*+/+*^ *p53* ^*−/−*^ offspring (for *Sirt7* ^*−/−*^ *p53* ^*−/−*^, 0.5%, down from the predicted 6.25%; for *Sirt7* ^*+/−*^ *p53* ^*−/−*^, 8.3% down from 12.5%, *p*-value <0.001 by χ^2^ test, Figure 1A). Together, these results indicate that p53 ablation not only does not rescue the embryonic lethality of SIRT7 mice but exacerbates it.

Given the embryonic lethality of *Sirt7* ^−/−^ *p53* ^−/−^ mice, we knocked down (KD) p53 in WT and *Sirt7^−/−^* MEFs by retroviral transduction (Figures 1B, C). WT and *Sirt7^−/−^* MEFs proliferate at similar rates (Vazquez et al., 2016), and p53 downregulation resulted in cellular immortalization of both MEFs genotypes as measured by their continuous proliferation overcoming replicative senescence. Interestingly, colony formation assays indicate that *Sirt*7^−/−^ cells downregulated for p53 (shRNA p53) display diminished cell growth based on the analysis of both colony number and size (Figure 1D). In addition, *Sirt7* ^−/−^ *p53 KD MEFs* (*Sirt7* ^−/−^shRNA p53) had higher apoptosis rates than wild type control MEFs (*Sirt7^+/+^* shRNA p53) (Figure 1E). Together, these data imply that in a p53-depleted cellular background, the loss of SIRT7 protein limits cellular growth and induces cell death.

### SIRT7 deficiency increases tumor formation in the absence of p53

Our previous reports (Vazquez et al., 2016) and current results (Supplementary Figure S1) indicate that SIRT7 knockout mice have a reduced life span compared to control mice, although the underlying cause of death, and whether it involves tumor formation, is uncertain. To address this issue, we performed gross anatomy analysis in 61 *Sirt7* ^*−/−*^mice in a 129/Sv genetic background in mice sacrificed when humane endpoints were reached, with a mean age of 12,3 months. Interestingly, megaesophagus, identified as a clear dilation of the esophagus, was the most prevalent pathology associated with *SirT7* ^*−/−*^ mice death, and no sign of tumors were found (Supplementary Figure S2). SIRT7 deficient mice in a C57/BL6 genetic background have severe embryonic lethality (Vazquez et al., 2016). Despite this embryonic lethality, a small cohort of *Sirt7* ^*−/−*^ mice survived to adulthood and aged to 11–15 months. Again, gross anatomy results showed no signs of tumorigenesis in these mice (data not shown). Because of the previous association of SIRT7 with the pathogenesis of cancer (Barber et al., 2012; Tang et al., 2017), this data indicates that the role of SIRT7 in cancer may depend on its interaction with other factors.

**FIGURE 2:**
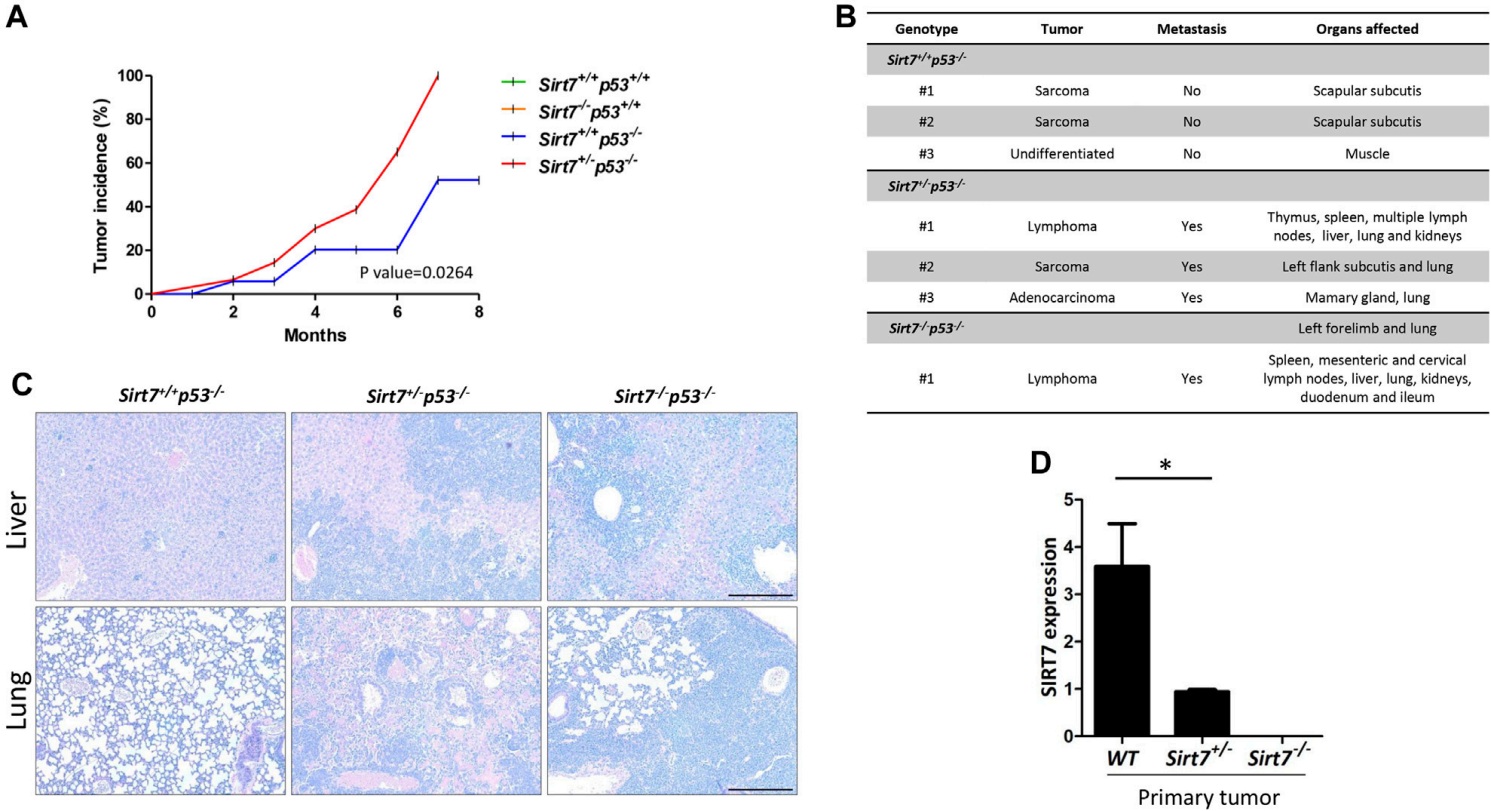
SIRT7 deficiency causes tumor formation in a p53-dependent manner associated with a metastatic phenotype. **(A)** Tumor incidence of different mice genotypes (*Sirt7* ^*+/+*^ *p53* ^*−/−*^ n = 20; *Sirt7* ^*+/−*^ *p53* ^*−/−*^ n = 16; *Sirt7* ^*+/+*^ *p53* ^*+/−*^ n = 38; *Sirt7* ^*−/−*^ *p53* ^*+/−*^ n = 11; *Sirt7* ^*+/+*^ *p53* ^*+/+*^ n = 12; *Sirt7* ^*−/−*^ *p53* ^*+/+*^ n = 4). **(B)** Histopathological analysis of tumor type, location, and spreading at the indicated mice genotypes. Representative images **(C)** and SIRT7 expression determined by RT-PCR **(D)** of primary tissue samples described in **(B)**. **(D)** Quantification represents the mean ± SEM of three experimental replicates from two tissue samples per indicated genotype. Data information: *p < 0.05 by *t*-test student.

*p53* ^*−/−*^ mice are born at normal Mendelian ratios. However, in adulthood, *p53* ^*−/−*^ mice develop a wide range of tumors, including lymphomas and sarcomas, and die early in life (Donehower, 1996). Therefore, we aimed to determine whether SIRT7 depletion could cooperate with p53 loss in tumor formation and development. Although concomitant depletion of SIRT7 and p53 was embryonic lethal in mice (Figure 1A), we analyzed the survival and tumor formation of other genetic combinations with particular emphasis on *Sirt7* ^*+/+*^ *p53* ^*−/−*^ and *Sirt7* ^*+/−*^ *p53* ^*−/−*^ mice. Kaplan-Meier analysis showed that *Sirt7* ^*+/−*^ *p53* ^*−/−*^ mice died slightly sooner than *Sirt7* ^*+/+*^ *p53* ^*−/−*^ mice, although not statistically significant (Supplementary Figure S1). Importantly, although both *Sirt7* ^*+/−*^ *p53* ^*−/−*^ and *Sirt7* ^*+/+*^ *p53* ^*−/−*^ mice developed spontaneous tumors starting at 2 months of age (Figure 2), *Sirt7* ^*+/−*^ *p53* ^*−/−*^ reached a 100% cumulative incidence by 7 months of age while at this age *Sirt7* ^*+/+*^ *p53* ^*−/−*^ only reached 50% (Figure 2A). The histopathological analysis to specify the type and location of tumors showed the development of secondary malignant growths indicative of metastasis in *Sirt7^+/−^ p53^−/−^* and *Sirt7^−/−^p53^−/−^* mice, including the liver and lungs, while in *Sirt7* ^+/+^ *p53* ^−/−^ mice no signs of metastasis were found (Figures 2B, C). Notably, SIRT7 expression remains in tumors from the *Sirt7* ^+/−^ *p53*−/− mice but statistically decreases compared with *Sirt7* ^+/+^ *p53* ^−/−^ mice samples (Figure 2D). We cannot discern whether these results indicated SIRT7 haploinsufficiency or loss of heterozygosity in *Sirt7* ^+/−^ tumor cells due to the heterogenicity of the samples. Specifically, we cannot ascertain the relative proportion of tumorigenic versus untransformed cells in these samples. Nevertheless, the results support that SIRT7 mRNA and plausible protein levels decrease in a *Sirt7* *^+/−^* tumor environment. Overall, our data indicates that in *p53* *^−/−^* mice, SIRT7 acts as a tumor suppressor and may protect against metastasis.

### SIRT7 levels predict patient outcome in some tumors with p53 mutations

**FIGURE 3:**
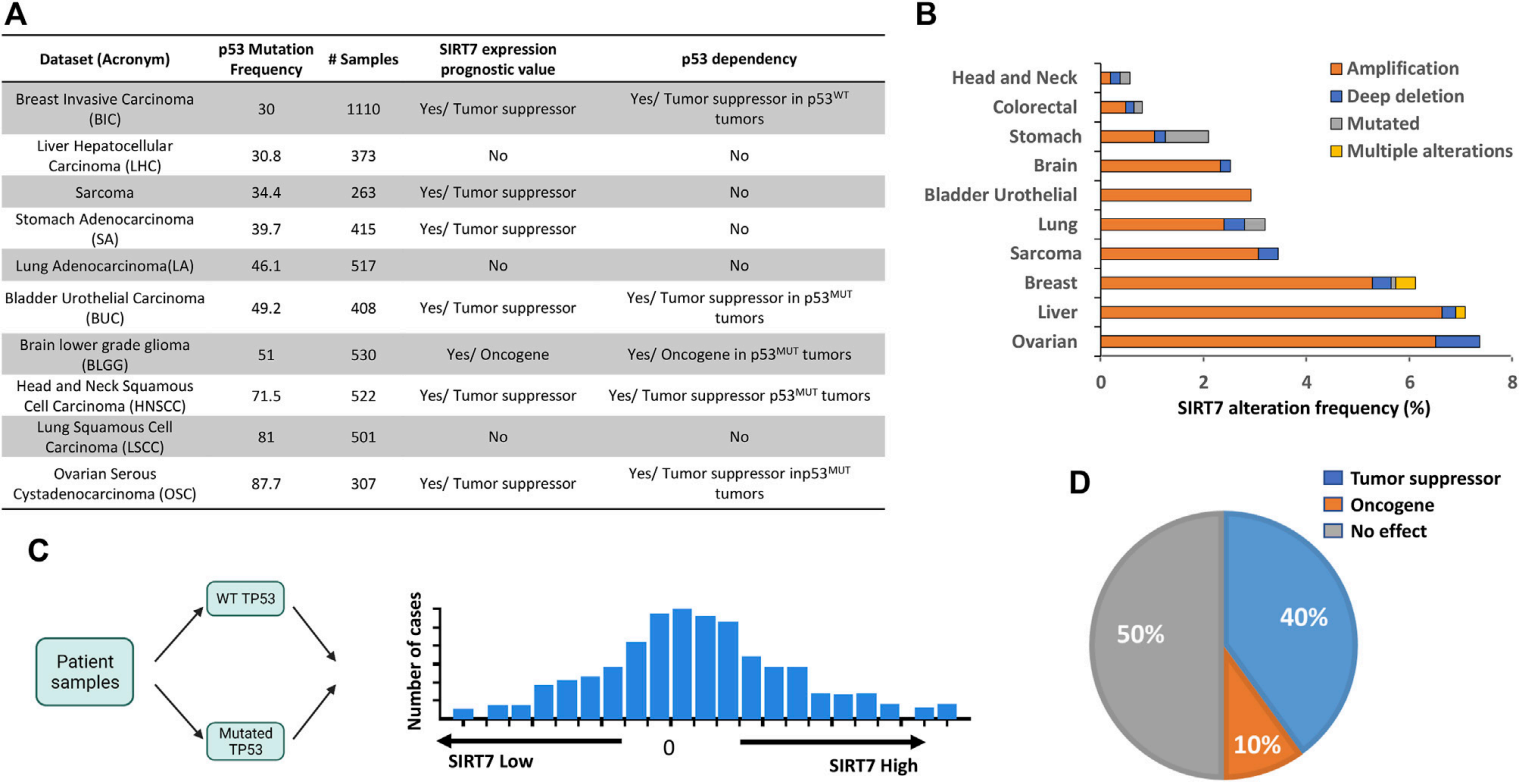
Deciphering the interplay between SIRT7 and p53 in cancer patients. **(A)** Table showing the list of tumor datasets, p53 mutation frequency (%), number samples with transcriptomics (RNA-Seq), and clinical data, including SIRT7 expression as a prognostic value and its dependency on p53 on patient overall survival **(B)** Mutation frequency and genetic alteration types in the SIRT7 gene at indicate cancer types **(C)** Scheme showing strategy to analyze SIRT7 and p53 interplay in human cancer. The tumor samples were initially categorized as either p53WT or p53MUT and subsequently divided into SIRT7^HIGH^ and SIRT7^Low^ groups based on binned expression profiles. **(D)** Pie chart illustrating the percentage of tumor samples where SIRT7 functions either as a tumor suppressor or an oncogene in a p53-dependent manner. N = 10 tumor datasets.

Based on the functional relationship observed between SIRT7 and p53 in tumor progression in our mouse model, our next objective was to explore whether this correlation holds in humans. Using the cBioportal Cancer Genomics portal (Cerami et al., 2012; Gao et al., 2013), we searched for cancer datasets with high p53 mutation frequency (over 30%) for which gene expression and clinical data were also available (Figure 3A). Interestingly, in these tumor datasets, the incidence of SIRT7 gene alterations was low, with SIRT7 gene amplification being the most prevalent form of mutation (Figure 3B). To investigate the association between SIRT7 and p53 in cancer prognosis, we initially categorized tumors based on p53 status, classifying them as p53 wild-type (p53^WT^) or mutant (p53^MUT^), and we further divided them into two groups based on the median score of SIRT7 mRNA expression: SIRT7 High (SIRT7^High^) and low (SIRT7^Low^) expression levels (Figure 3C).

**FIGURE 4:**
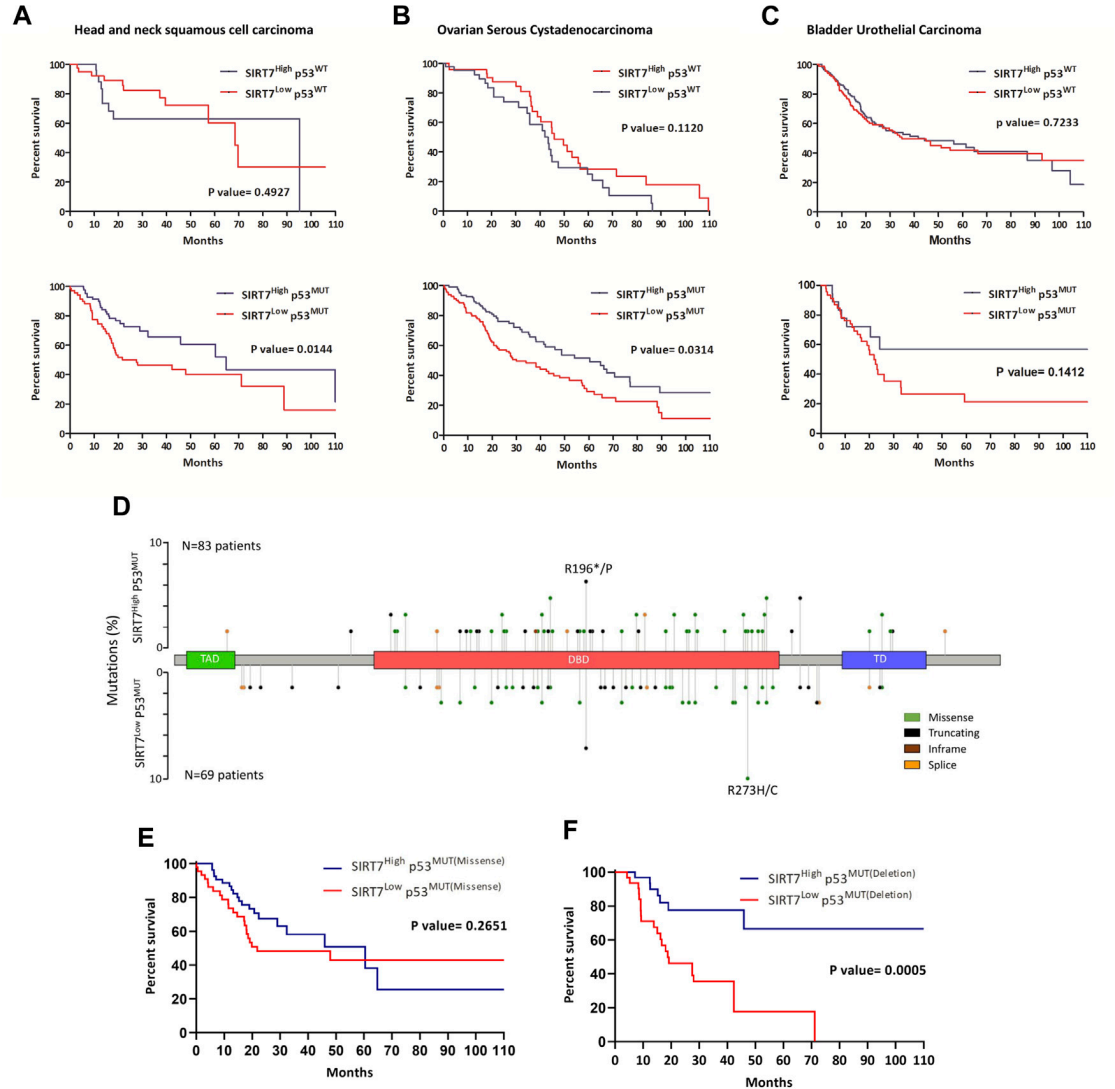
SIRT7 acts as a tumor suppressor in a p53-dependent manner in specific human tumors. **(A–D)** Kaplan–Meier survival curves of patients with a diagnosis of head and neck squamous cell carcinoma **(A,D)**, ovarian serous cystadenocarcinoma **(B)**, and bladder urothelial carcinoma **(C)** based on SIRT7 expression levels and p53 mutations. HNSCC, N = 26 and N = 34 for SIRT7^HIGH^ and SIRT7^LOW^, respectively in p53^WT^ tumors, N = 82 and N = 69 in SIRT7^HIGH^ and SIRT7^LOW^ respectively in p53^MUT^ tumors; OSAC, N = 47 and N = 44 for SIRT7^HIGH^ and SIRT7^LOW^ in p53^WT^ tumors, N = 46 and N = 46 in SIRT7^HIGH^ and SIRT7^LOW^ in p53^MUT^ tumors; BUC, N = 182 and N = 159 for SIRT7^HIGH^ and SIRT7^LOW^ in p53^WT^ tumors, N = 18 and N = 46 in SIRT7^HIGH^ and SIRT7^LOW^ in p53^MUT^ tumors. **(D)** Lollipop plot of p53 gene mutations in head and neck cancer. *Y*-axis denotes percentages of patients harboring p53 mutations in SIRT7^High^ and SIRT7^Low^ groups. TAD: transactivation domain; DBD: DNA binding domain; TD: Tetramerization domain. **(E,F)** Kaplan–Meier survival curves of patients with a diagnosis of head and neck squamous cell carcinoma grouped based on p53 mutation type, whether missense or deleting mutations. In patients with p53 missense mutacions, N = 62 and N = 45 for SIRT7^HIGH^ and SIRT7^LOW^ tumors. In patients harboring p53 deleting mutations, N = 33 and N = 3 for SIRT7^HIGH^ and SIRT7^LOW^ tumors. Data information: *p*-value determined by a two-sided log-rank test (Mantel-Cox).

To evaluate the correlation of SIRT7 expression with clinical outcomes, we compared the SIRT7^High^ and SIRT7^Low^ groups. Our analysis of patient overall survival revealed that low SIRT7 mRNA levels correlate with worse overall survival in 60% of patients, suggesting a potential tumor suppressor role of SIRT7 in this type of tumors, regardless of p53 status (Figure 3A). We next examined whether SIRT7 acts in a p53-dependent manner by comparing patient overall survival in p53^WT^ and p53^MUT^ datasets. When considering the p53 dependency, we observed that SIRT7 behaved as a tumor suppressor in 40% and as a tumor promoter in 10% of the cases (Figures 3A, D). Kaplan-Meier analysis demonstrated that in breast invasive carcinoma (BIC), SIRT7 acts as a tumor suppressor only in p53^WT^ samples and in brain low-grade glioma (BLGG) as an oncogene only in p53^MUT^ tumors (Supplementary Figures S3A, S3B, respectively). However, in head and neck squamous cell carcinoma (HNSCC), ovarian serous cystadenocarcinoma (OSC), and bladder urothelial carcinoma (BUC), SIRT7 low expression levels dramatically reduce the median survival time in p53^MUT^ tumors, but not in p53^WT^ tumors (Figure 4). For patients with HNSCC cancer, the median survival was 64 months for the SIRT7^High^p53^MUT^ group and 27 months for the SIRT7^Low^p53^MUT^ group (Figure 4A). Similarly, in patients with ovarian cancer carrying p53 mutations, the median survival time was 60 weeks for tumors with high levels of SIRT7 and 30 weeks for tumors with low levels of SIRT7 (Figure 4B). Finally, in bladder urothelial carcinoma, the median survival time for the SIRT7^Low^p53^MUT^ is reduced from 60 to 20 months (Figure 4C). Consistent with previous reports (Kennedy and Lowe, 2022), p53 mutational analysis in head and neck cancer revealed that p53 mutations mainly target the DNA binding domain, encompassing missense and deletion mutations (Figure 4D). While p53 deletion mutations contribute to tumorigenesis through p53 loss alone, p53 missense mutations not only alter p53 ability to bind DNA and activate downstream target genes but can also promote gain-of-function activities by interacting with novel partners. Notably, patient survival analysis considering the p53 mutation type (Figures 4E, F) revealed that survival rates among patients harboring p53 missense mutations were similar between SIRT7^High^ and SIRT7^Low^ groups. However, in the case of patients with p53 putative deletion mutations, the SIRT7^Low^ group displayed a notably reduced survival rate. These results suggest that, in human cancers, SIRT7’s role in tumor progression is both cancer type and p53-status dependent. Except for BLGG, when SIRT7 acts as a tumor suppressor in p53 mutated tumors, its low expression may predict unfavorable cancer prognosis.

### Synergistic effect of SIRT7 and p53 on extracellular matrix gene expression regulation in tumor and embryonic cells

**FIGURE 5:**
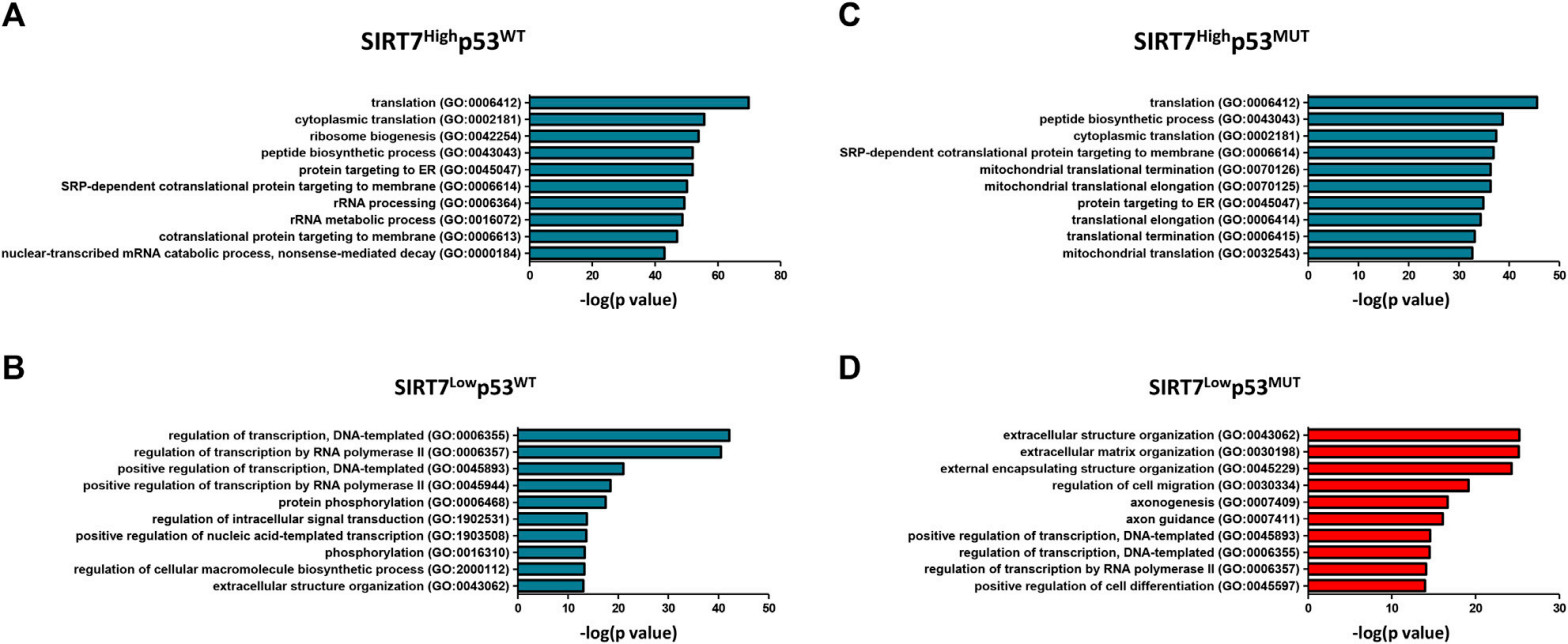
Gene ontology of SIRT7 associated genes in WT and p53 mutant tumors. **(A–D)** Bar plots showing top ten GO terms (biological process) of upregulated genes in Head and neck squamous cell carcinoma. Left plots **(A–B)** correspond to datasets in p53^WT^ of SIRT7^HIGH^ **(A)** and SIRT7^LOW^ **(B)** tumors, and right plots **(C–D)** correspond to datasets in p53^MUT^ of SIRT7^HIGH^ **(C)** and SIRT7^LOW^ **(D)** tumors.

To further understand SIRT7-p53 interplay as tumor suppressors, we obtained normalized expression data on significantly upregulated genes (adjusted *p*-value <0.05) from HNSCC, BUC, and OSC tumor datasets and performed gene ontology (GO) analysis (Figure 5, Supplementary Figures S4, S5). In tumors with low SIRT7 levels and intact p53, and consistent with previous observations on the role of SIRT7 in gene expression (Blank and Grummt, 2017), there was an enrichment in biological processes related to gene transcription regulation (Figure 5B; Supplementary Figures S4B, S5B). Interestingly, bladder and head and neck cancers with low SIRT7 levels and mutated p53 gene ontology analysis revealed biological processes related to extracellular matrix organization (Figure 5D, Supplementary Figure S4D). In ovarian cancer samples (Supplementary Figure S5D), a GO term related to cell-matrix adhesion was also present in addition to other biological processes such as cell differentiation and metabolism. The imbalanced expression of extracellular matrix proteins significantly contributes to cancer dissemination and the acquisition of metastatic characteristics (Winkler et al., 2020). Hence, our findings suggest a crucial role for SIRT7 in cancer dissemination in a p53-dependent manner. These findings align with previous studies demonstrating that SIRT7 limits TGF-β1 signaling to prevent metastasis in breast cancer cells (Tang et al., 2017). Indeed, a survival analysis conducted on breast cancer patients using the Cbioportal database reveals a significant correlation between low expression of SIRT7 and unfavorable patient outcomes, supporting a role of SIRT7 as a tumor suppressor in this cancer type (Supplementary Figure S3A). However, SIRT7 expression is positively correlated with patient survival exclusively in p53^WT^ breast cancer cells, while no prognostic value for SIRT7 is present in p53^MUT^ tumoral cells. This finding suggests that SIRT7 may have distinct roles in regulating cancer dissemination in breast cancer cells compared to head and neck, and bladder cancer cells.

**FIGURE 6:**
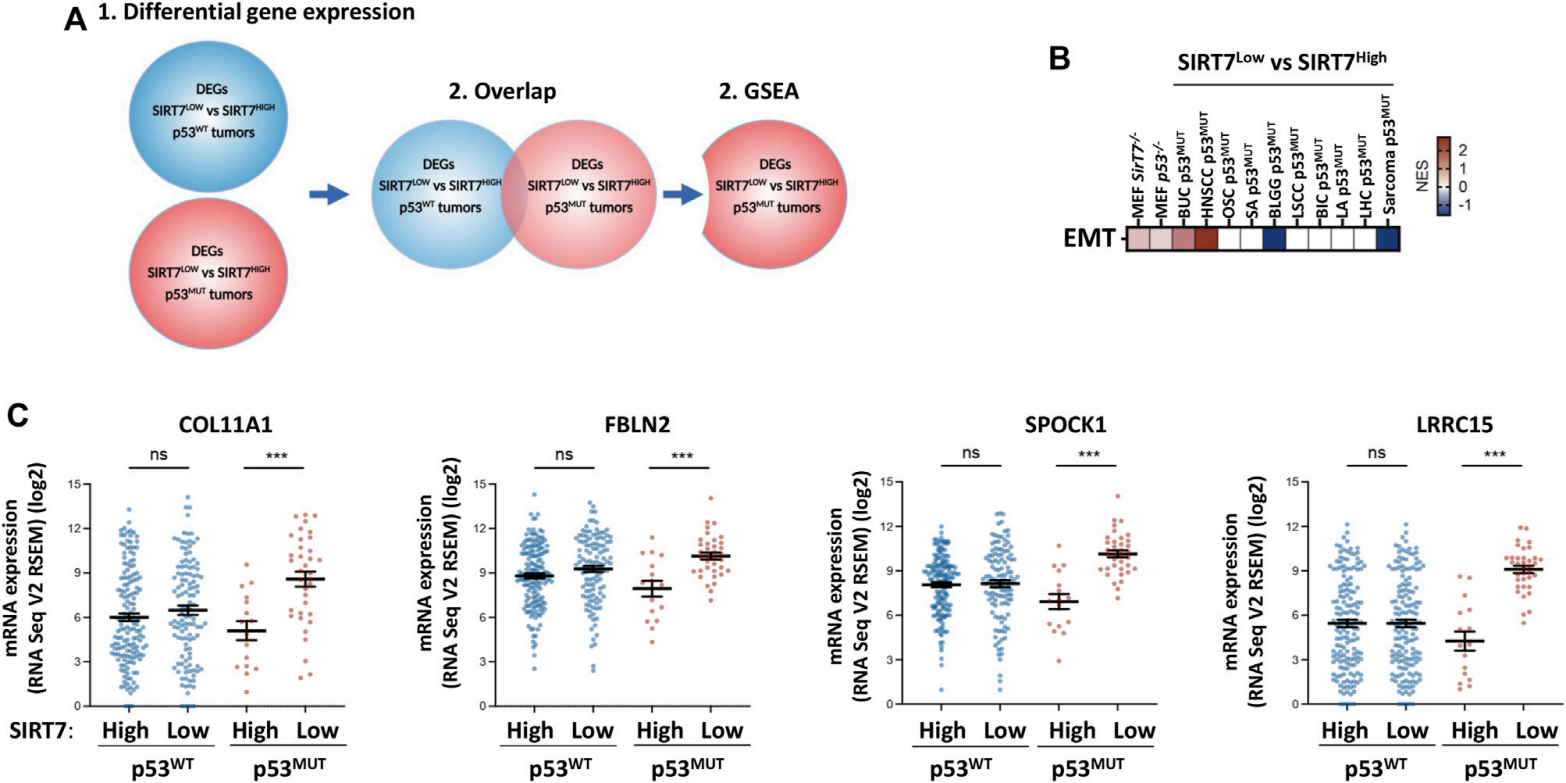
Low SIRT7 expression in p53 mutant tumors results in deregulation of EMT pathways **(A)** Schematic diagram of the approach used to identify subsets of genes regulated by SIRT7 exclusively in p53 mutant tumors. Differential gene expression analysis was performed independently in p53 WT and mutant samples comparing the transcriptomes of tumors with low vs. high SIRT7 mRNA expression. An overlap analysis was then performed to identify genes exclusively differentially expressed between SIRT7 expression groups only in p53 mutant samples. **(B)** Gene set enrichment analysis of genes identified as shown in **(A)**. The graph shows the enrichment of genes involved in epithelial-mesenchymal transition (EMT) in specific tumor types with p53 mutations depending on SIRT7 expression. NES: Normalized Enrichment Score. Red: enriched in patients with low SIRT7. Blue: enriched in patients with high SIRT7. White: no enrichment. **(C)** mRNA expression of several EMT genes (COL11A1, FBLN2, SPOCK1 and LRCC15) in the indicated groups of BUC tumors.

To unambiguously identify specific molecular signatures associated with the concomitant loss of SIRT7 and p53, we also adopted an unbiased approach with all tumor datasets independent of the clinical outcome (Figure 6). For each type of tumor, we performed differential expression gene (DEG) analysis between tumors expressing high or low SIRT7 in the context of p53^WT^ or p53^MUT^ (Figure 6A). Next, we overlapped SIRT7-dependent DEGs from WT and mutant p53 and excluded the genes that showed common deregulation across all conditions. By doing this, we selected SIRT7-dependent DEGs with altered expression only in p53 mutant tumors and thus potential targets of a specific function of SIRT7 in p53 mutant tumors. Accounting for both gene interactions and gene expression fold changes, we subsequently conducted Gene Set Enrichment Analysis (GSEA) on these genes. Notably, we observed a significant enrichment of the EMT signature, specifically in p53 mutant tumors with low SIRT7 expression for both BUC and HNSCC (Figure 6B). Amongst the EMT genes clearly differentially expressed depending on SIRT7 expression in p53^MUT^ but not p53^WT^ BUC tumors were collagen type XI alpha-1 (COL11A1), Fibulin 2 (FBLN2), SPOCK1 and Leucine Rich Repeat Containing 15 (LRCC15), genes with major roles in extracellular matrix regulation and with a previously described oncogenic potential in BUC (Figure 6C) (Ma et al., 2016; Li et al., 2020; Liu et al., 2021). The remaining tumor types did not present EMT pathway enrichment, except in BLGG and sarcoma, where the EMT signature is present in tumors with mutated p53 and high SIRT7 expression (Figure 6B). Overall and consistent with our GO results (Figure 5, Supplementary Figures S4, S5), our findings underscore the crucial involvement of SIRT7 in regulating EMT pathway, specifically in HNSCC and BUC tumors, in a p53-dependent manner. This observation highlights the potential significance of SIRT7 in inhibiting cancer aggressiveness and impeding disease progression in these tumor types.

### SIRT7 and p53 co-depletion increases cellular migration in immortalized MEFs

**FIGURE 7:**
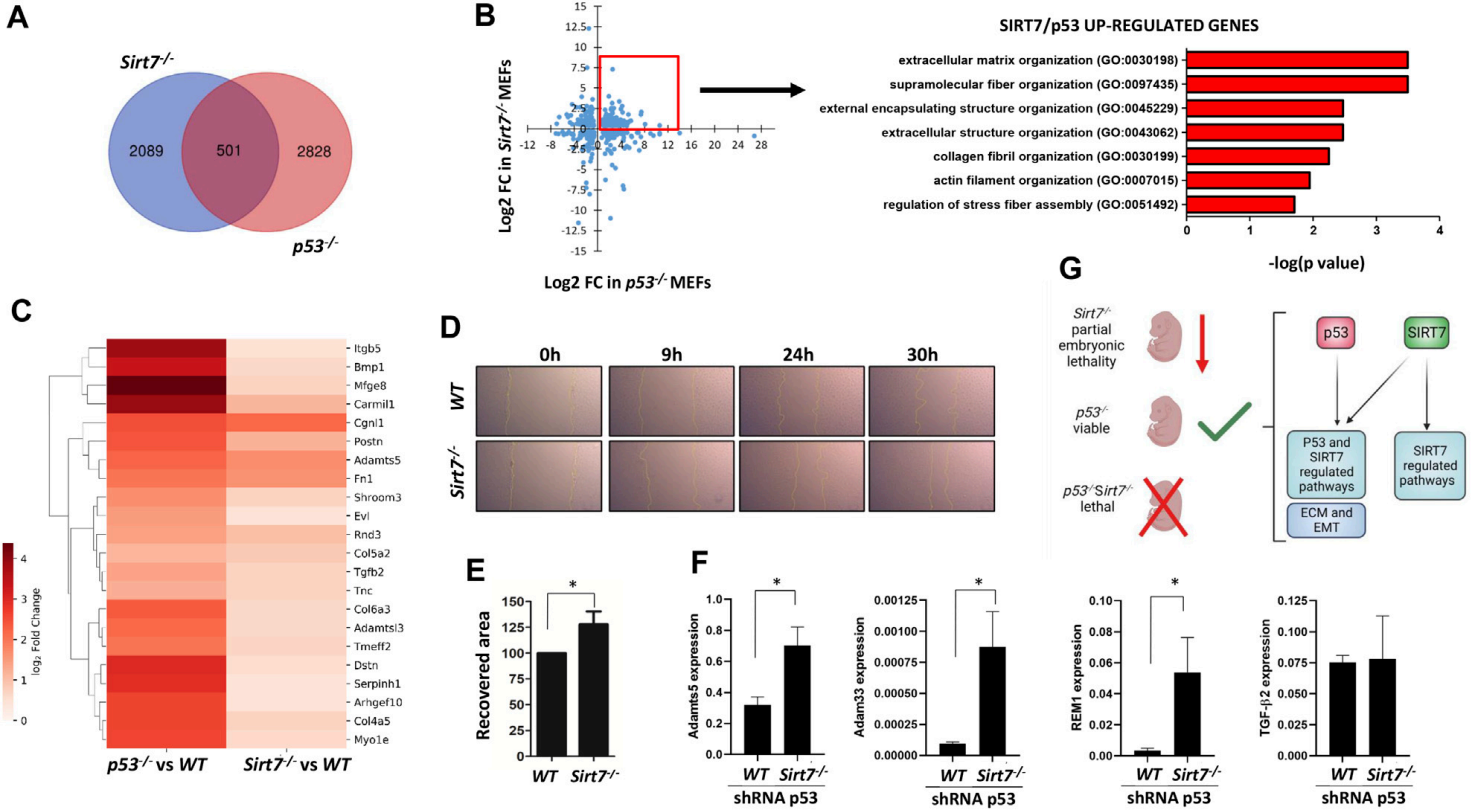
SIRT7 and p53 negatively regulate genes related with extracellular matrix organization in mouse embryonic fibroblasts. **(A)** Venn diagram depicting the overlap between DEGs in *Sirt7* ^*−/−*^ and *p53* ^*−/−*^ MEFs. **(B)** Scatter plot of common DEGs (left) and GO analysis of common upregulated genes (right) in *Sirt7* ^*−/−*^ and *p53* ^*−/−*^ MEFs. **(C)** Heatmap of differentially expressed genes in *Sirt7* ^*−/−*^ and *p53* ^*−/−*^ MEFs from the extracellular matrix regulation GO term. **(D–E)** Wounding assay on WT and *Sirt7* ^***−/−***^ MEFs shRNA for p53. Representative pictures of wound closure at 9h, 24 and 30 h. Quantification of the percent wound closure of cells at 30 h post wound injury. **(F)** RT-PCR analysis of WT and *Sirt7* ^*−/−*^ MEFs shRNA for p53 of indicated genes. Quantification represents the mean ± SEM of three independent experiments conducted in three independent cell lines. Data information: *p < 0.05 by *t*-test. **(G)** The distinct embryonic viability observed in Sirt7, p53, and double mutant mice suggests the model of an overlapping regulation of genes involved in ECM and EMT pathways by both proteins, which gets dysfunctional upon the loss of both genes. ECM: Extracellular matrix, EMT: epithelial-to-mesenchymal transition.

EMT regulation during embryogenesis is chief for tissue remodeling and organogenesis by regulating cellular adhesion and migration, among other processes (Campbell, 2018). Therefore, we interrogated whether SIRT7 and p53 could also coregulate these pathways in embryonic cells (Figure 7). We used publicly available RNA-seq data on WT, *Sirt7* ^*−/−*^ and *p53* ^*−/−*^ unstressed MEFs (Kenzelmann Broz et al., 2013; Simonet et al., 2020) in our GSEA analysis and, of note, DEGs in *Sirt7* ^*−/−*^ and *p53* ^*−/−*^ MEFs also show a positive enrichment for the EMT signature (Figure 6B). Compared to WT cells, there were 2590 DEGs in Sirt7^−/−^ cells and 3329 DEGs in *p53* ^*−/−*^ cells (*p*-value > 0.05 and fold change >2) (Figure 7A). Interestingly, we found 501 genes commonly deregulated in *Sirt7* ^*−/−*^ and *p53* ^*−/−*^ deficient cells. Like what we observed in some tumors (Figure 5, Supplementary Figures S4, S5), GO analysis unveiled that genes upregulated in both Sirt7 and p53 deficient cells belong to biological processes related with extracellular matrix organization and regulation (Figures 7C, D), while commonly downregulated genes related with RNA biology and translation (Supplementary Figure S6). These results support that SIRT7 and p53 functionally overlap in controlling the balance between cellular proliferation and EMT in MEFs, potentially impacting tissue morphogenesis and overall embryo development. To further analyze the role of SIRT7 and p53 in early development, we knock down SIRT7 and p53 expression in mouse ESCs and induce EB differentiation over a 5 day period (Supplementary Figure S7). Even though all conditions led to EB formation, we observed an altered expression pattern of early germline markers, suggesting an essential role of both SIRT7 and p53 in early cell fate specification.

Since cellular migration is an important feature of cellular undergoing EMT (Yang et al., 2020), we conducted a scratch wound healing assay to assess the migratory capacity of WT and *Sirt7* ^*−/−*^ p53-KD cells. Notably, *Sirt7* ^*−/−*^ p53-KD cells exhibited a significantly faster refilling of wounded empty spaces compared to WT p53-KD control cells at the 30-h time point (Figures 7D, E).

Among the genes involved in EMT and cell-matrix biology that showed dysregulation in the transcriptome of *Sirt7* ^*−/−*^ and *p53* ^*−/−*^ MEFs (Kenzelmann Broz et al., 2013; Simonet et al., 2020), there was a significant upregulation of ADAM Metallopeptidase With Thrombospondin Type 1 Motif 5 (Adamts5)**,** ADAM Metallopeptidase Domain 33 (Adam33), as well as RRAD And GEM Like GTPase 1 (REM1) (Figure 7F), essential in cytoskeletal reorganization and cell-matrix interactions (Edwards et al., 2008; Rose et al., 2021) in *Sirt7* ^*−/−*^ MEFs p53-KD. However, the expression of Transforming Growth Factor Beta 2 (TGFβ2), a key regulator of extracellular matrix remodeling (Xie et al., 2018), remained similar between WT and *Sirt7* ^*−/−*^ MEFs with p53-KD. Overall, these results collectively suggest that concomitant decreased levels of SIRT7 and p53 lead to enhanced cellular migration, which could result from gene expression alterations of extracellular matrix genes.

## Discussion

The results reported here indicate that concomitant SIRT7 and p53 depletion negatively impact embryo development as the absence of p53 intensifies the embryonic lethality observed in *Sirt7* ^*−/−*^ mice (Figure 1A). These results suggest that p53 activity is required to sustain development in the absence of SIRT7. To our knowledge, this is the first Sirtuin reported to interact functionally with p53 during embryo development. *Sirt1* ^*−/−*^ mice have severe embryonic lethality independent of p53 activity (Kamel et al., 2006), and *Sirt6* ^*−/−*^ mice have normal embryo development (Mostoslavsky et al., 2006). Interestingly, *Sirt6* ^*−/−*^ mice die early after birth partially due to p53 hyperactivation (Ghosh et al., 2018). Together, these observations suggest that the interaction between different Sirtuins and p53 takes place, orchestrated in a development stage-specific manner.

Further reinforcing the interplay between SIRT7 and p53 in embryo development, analysis of DEGs in MEFs derived from *Sirt7* ^*−/−*^ and *P53* ^*−/−*^ mice indicated a substantial overlap between both genotypes (Figure 7). In addition, GO and GSEA analysis revealed enrichment of pathways related to EMT, extracellular matrix, and rRNA biology (Figure 6B; Figure 7B; Supplementary Figure S6), central to sustaining embryonic tissue morphogenesis and growth. EMT processes involve the cellular loss of epithelial cellular traits, acquisition of migratory capabilities and gain of mesenchymal markers. Interestingly, our results indicate that SIRT7 deficiency increases the migration capabilities of p53-KD MEFs and alters the expression of genes related to cytoskeleton organization and cell-matrix interactions (Figures 7D–F). Both SIRT7 and p53 were previously associated with extracellular matrix remodeling during tissue fibrosis (Wyman et al., 2017; Wu et al., 2020; Yu et al., 2022), but whether this function may be important in other situations is unknown. Because EMT is an essential process in normal embryonic development and organogenesis, our results suggest that the interplay of SIRT7 and p53 in embryonic cells may be important to sustain tissue morphogenesis.

p53-deficient mice develop spontaneous tumors early in life and constitute a feasible model to study cancer-promoting and suppressive capabilities of other factors (Donehower, 1996). In this sense, previous studies showed that both SIRT1 and SIRT6 limit tumor onset and development in p53 deficient mouse models (Wang et al., 2008; Ghosh et al., 2018), uncovering the important tumor suppressor functions of these Sirtuins. Similarly, our results indicate that SIRT7 acts as a tumor suppressor in p53-deficient mice. Importantly, *Sirt7* ^*+/−*^ *p53* ^*−/−*^ mice, compared to *Sirt7* ^*+/+*^ *p53* ^*−/−*^ mice, present a significantly higher tumor formation (Figure 2A) and a more aggressive cancer progression as histopathological analysis revealed tumor dissemination to different organs (Figure 2B). SIRT7 is frequently overexpressed in multiple cancer types and has generally been considered an oncogene (Barber et al., 2012; Paredes et al., 2014; Malik et al., 2015). However, the use of cancer-specific mouse models and the availability of patient survival data have unveiled both oncogenic and tumor suppressive properties of SIRT7 (Tang et al., 2017). Our results support the notion that the role of SIRT7 as a tumor suppressor may depend on other factors, and as demonstrated in this study (Figure 2), including p53-dependency. Neither *Sirt7* *^−/−^* mice from our vast 129Sv colony (Vazquez et al., 2016) nor the Sirt7^−/−^ mice of this study developed tumors (Supplementary Figure S2). Although, it is true that *Sirt7* ^*−/−*^ mice indeed present phenotypic and molecular signs of severe accelerated aging that contribute to their reduced lifespan (Vazquez et al., 2016) and could, in addition, preclude the dissemination of oncogenic cells.

In humans, we have identified three cancer types (BUC, HNSCC, OSC) where low SIRT7 levels, combined with p53 mutations, significantly correlate with poor disease prognosis. Interestingly, GSEA analysis revealed that SIRT7-dependent DEGs in these cancer types were associated with EMT pathways, suggesting that poor patient survival in these datasets results from enhanced cancer dissemination, similar of what we observed in mice (Figure 2). A previous report associated SIRT7 with inhibition of tumor metastasis in a mouse model of breast cancer (Tang et al., 2017). In this study, SIRT7 limited cell migration and invasiveness through the control of SMAD4 protein activity, a major downstream effector of TGFβ1 signaling and related to EMT. However, our analysis indicates that in breast cancer patients, SIRT7 expression serves as a prognostic value in tumors harboring WT but not mutated p53, highlighting a complex interplay between SIRT7 and p53 across different cancer types. Nevertheless, because *Sirt7* ^*+/−*^ *p53* ^*−/−*^ mice had higher tumor incidence but similar tumor onset compared to *Sirt7* ^*+/+*^ *p53* ^*−/−*^ (Figure 2)*,* our results indicate an important role in tumor growth rather than tumor initiation.

Sirtuin function and p53 regulation and signaling are critically linked. A prominent example is the interplay between SIRT6 and p53, where SIRT6 restricts p53 stability and activity through deacetylation at K381 (Ghosh et al., 2018). SIRT1, on the other hand, deacetylates lysine 382 of p53, but the functional consequences of the SIRT1-p53 interplay remain unclear due to conflicting reports of both p53 activation and inhibition upon SIRT1 overexpression (Langley et al., 2002; Cheng et al., 2003; Kamel et al., 2006). Despite initial studies on the interplay between SIRT7 and p53 described an inhibitory role for SIRT7 on p53 activity through lysine deacetylation (Vakhrusheva et al., 2008), increasing evidence indicates a more complex functional interplay (Kumari et al., 2021). One of the current models is that under favorable conditions, SIRT7 ensures p53 low activity, and upon genotoxic stress, however, SIRT7 activity switches to promote p53 stabilization and accumulation through indirect mechanisms (Ianni et al., 2021). In addition to the classical p53-mediated stress response, our study suggests that SIRT7 and p53 functional interplay is involved in molecular pathways important for normal embryogenesis and abnormally reactivated in cancer. p53 and SIRT7 may bind the same gene promoters to co-repress gene expression. The intriguing observation that *Sirt7* ^*−/−*^ embryos experience partial embryonic lethality while *p53* ^*−/−*^ embryos develop normally, yet *Sirt7* ^*−/−*^ *p53* ^*−/−*^ embryos are nonviable, however, raises the possibility that p53 and SIRT7 may target through distinct pathways an overlapping set of genes, suggesting the model depicted in Figure 7G. Future investigations should provide insight into the molecular mechanisms underlying this novel functional interplay and its potential implication in developmental-scheduled or aberrant cellular morphogenesis.

## Data Availability

The datasets presented in this study can be found in online repositories. The names of the repository/repositories and accession number(s) can be found in the article/Supplementary Material.

## Supplementary material

SUPPLEMENTARY FIGURE S1 Kaplan Meier survival curve of *Sirt7* ^*+/+*^ *p53* ^*+/+*^ (green, n=13), *Sirt7* ^*-/-*^ *p53* ^*+/+*^ (yellow, n=7), *Sirt7* ^*+/+*^ *p53* ^*-/-*^ (blue, n=20) and *Sirt7* ^*+/-*^ *p53* ^*-/-*^ (red, n=16) mice.

SUPPLEMENTARY FIGURE S2 Gross anatomy results and cause of death o *Sirt7* ^*-/-*^ mice. Mice were subjected to a postmortem examination as described in the material and methods section.

SUPPLEMENTARY FIGURE S3 Deciphering the interplay between SIRT7 and p53 in human cancer. Kaplan-Meier survival curves illustrate the survival outcomes of patients diagnosed with breast invasive carcinoma (A) and brain lower-grade glioma (B). BIC, N=119 and N=118 for SIRT7^HIGH^ and SIRT7^LOW^ respectively in p53^WT^ tumors, N=55 and N=35 in SIRT7^HIGH^ and SIRT7^LOW^ respectively in p53^MUT^ tumors; BLGG, N=174 and N=166 for SIRT7^HIGH^ and SIRT7^LOW^ respectively in p53^WT^ tumors, N=50 and N=96 in SIRT7^HIGH^ and SIRT7^LOW^ respectively in p53^MUT^ tumors.

SUPPLEMENTARY FIGURE S4 Gene ontology of SIRT7-associated genes in WT and p53 mutant bladder urothelial tumors. **(A-D)** Bar plots showing the top ten GO terms (biological process) of upregulated genes in the indicated genotypes. **(A-B)** correspond to datasets of SIRT7^HIGH^ **(A)** and SIRT7^LOW^ **(B)** in p53^WT^ tumors, and **(C-D)** correspond to datasets of SIRT7^HIGH^ **(C)** and SIRT7^LOW^ **(D)** in p53^MUT^ tumors.

SUPPLEMENTARY FIGURE S5 Gene ontology of SIRT7-associated genes in WT and p53 mutant Ovarian Serous Cystadenocarcinoma tumors. **(A-D)** Bar plots showing the top ten GO terms (biological process) of upregulated genes in the indicated genotypes. **(A-B)** correspond to datasets of SIRT7^HIGH^ **(A)** and SIRT7^LOW^ **(B)** in p53^WT^ tumors, and** (C-D)** correspond to datasets of SIRT7^HIGH^ **(C)** and SIRT7^LOW^ **(D)** in p53^MUT^ tumors.

SUPPLEMENTARY FIGURE S6 Gene ontology of SIRT7 and p53 overlapping transcriptomes. Bar plots showing the top ten GO terms (biological process) of common DEGs in *Sirt7* ^*-/-*^ and *p53* ^*-/-*^ cells, including commonly upregulated genes **(A)**, downregulated **(B)**, upregulated and downregulated in *Sirt7* ^−/−^ and *p53* ^*-/-*^ respectively **(C)** and vice versa **(D)**.

SUPPLEMENTARY FIGURE S7 SIRT7 and p53 participate in early germline specification. **(A)** Images of 5-day EBs derived from ESCs transfected with SCR, Sirt7, p53, or Sirt7/p53 shRNAs **(B-C)**. RT-PCR analysis of GATA-4, GATA-6 (endo/mesoderm markers) and PAX6 and Nestin. Quantification represents the mean ± SEM. Data information: **P < 0.05; **P < 0.01; ***P < 0.001; by t-test.
