# Supplementary figures and images for: SIRT7 and p53 interaction in embryonic development and tumorigenesis
Source: Front Cell Dev Biol. 2024 Jan 3;11:1281730. doi: 10.3389/fcell.2023.1281730 (PMC10791984; doi:10.3389/fcell.2023.1281730)

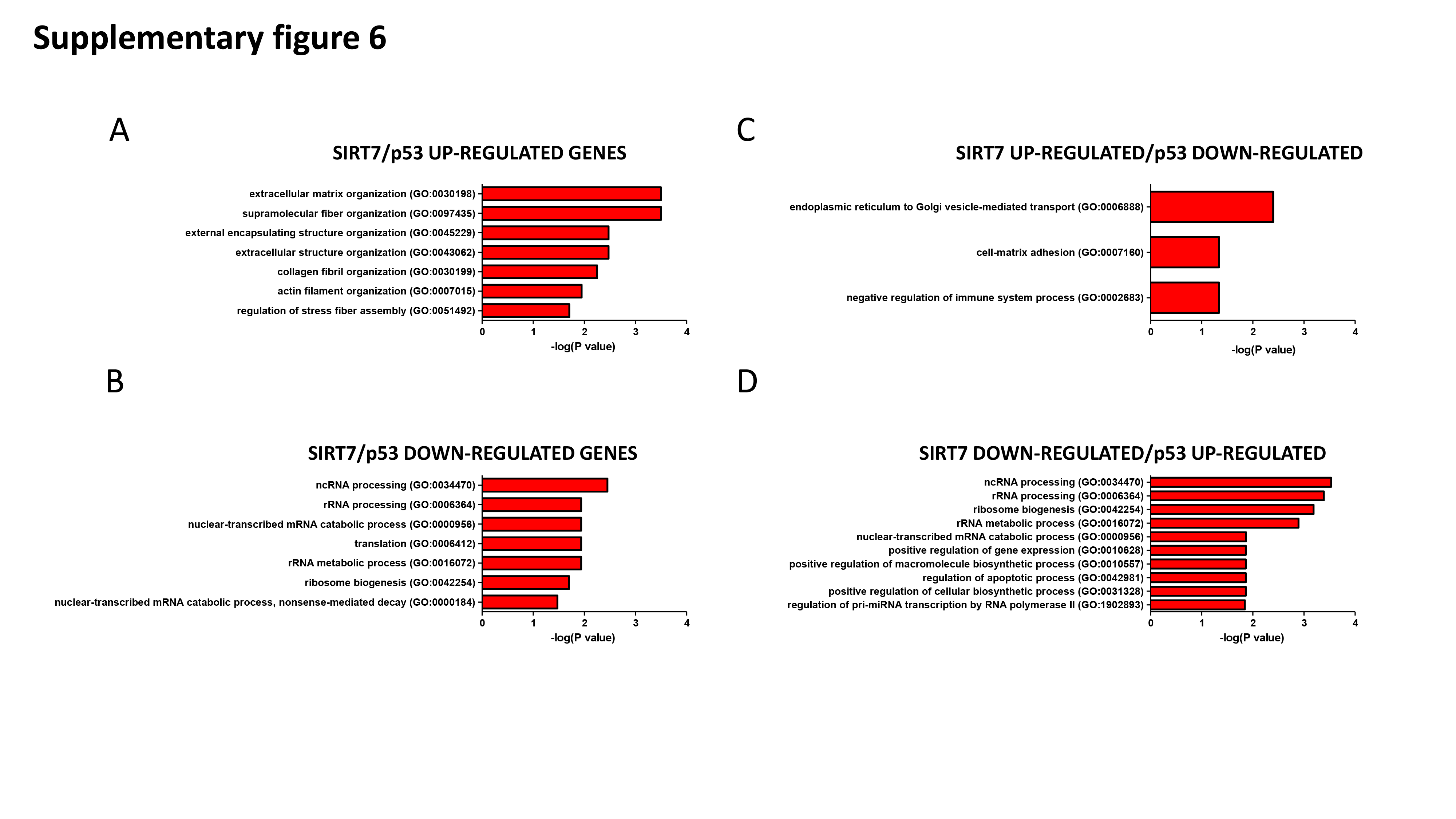

Supplement: Supplementary file 1 [file Image6.TIF]

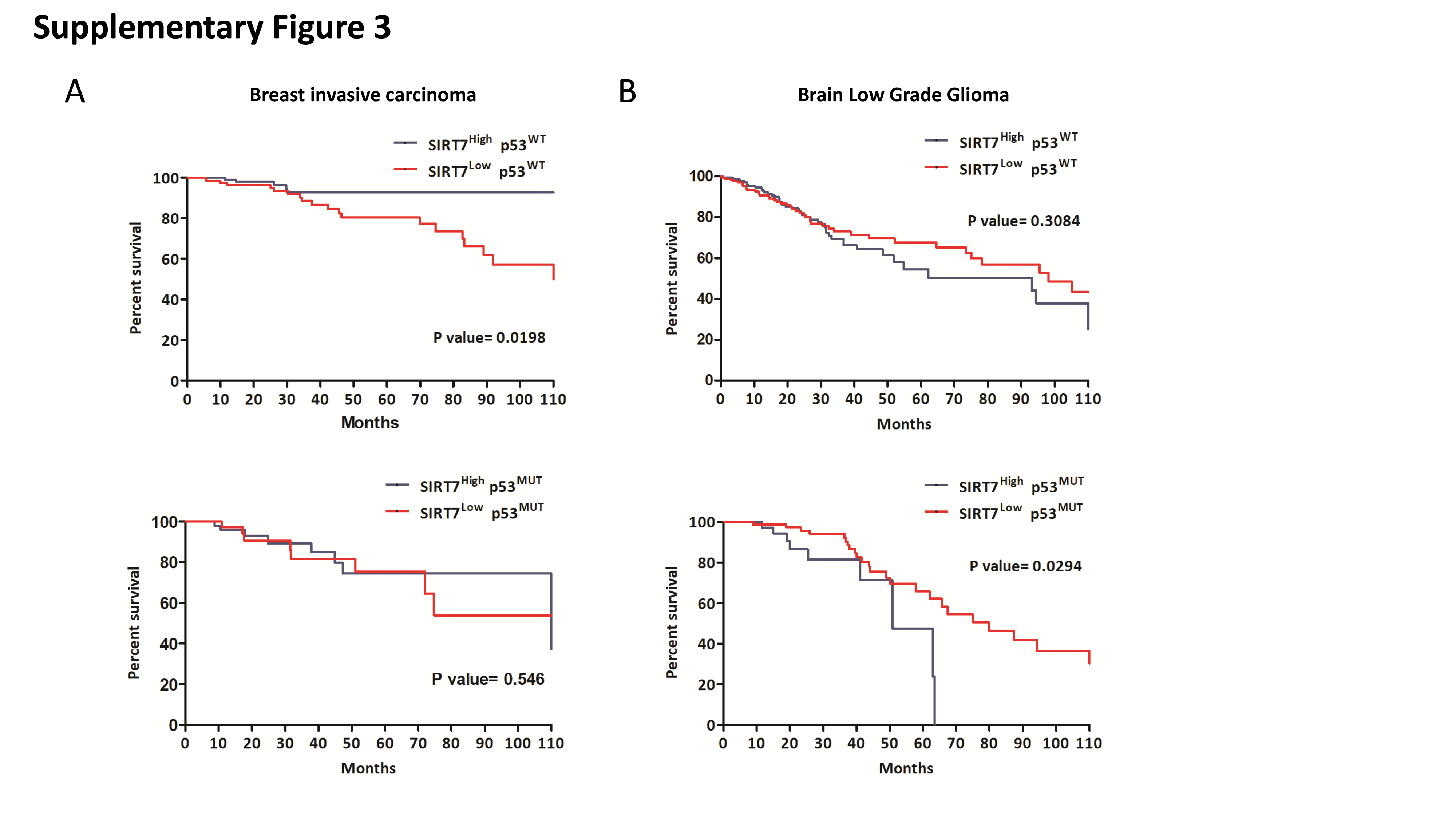

Supplement: Supplementary file 2 [file Image3.TIF]

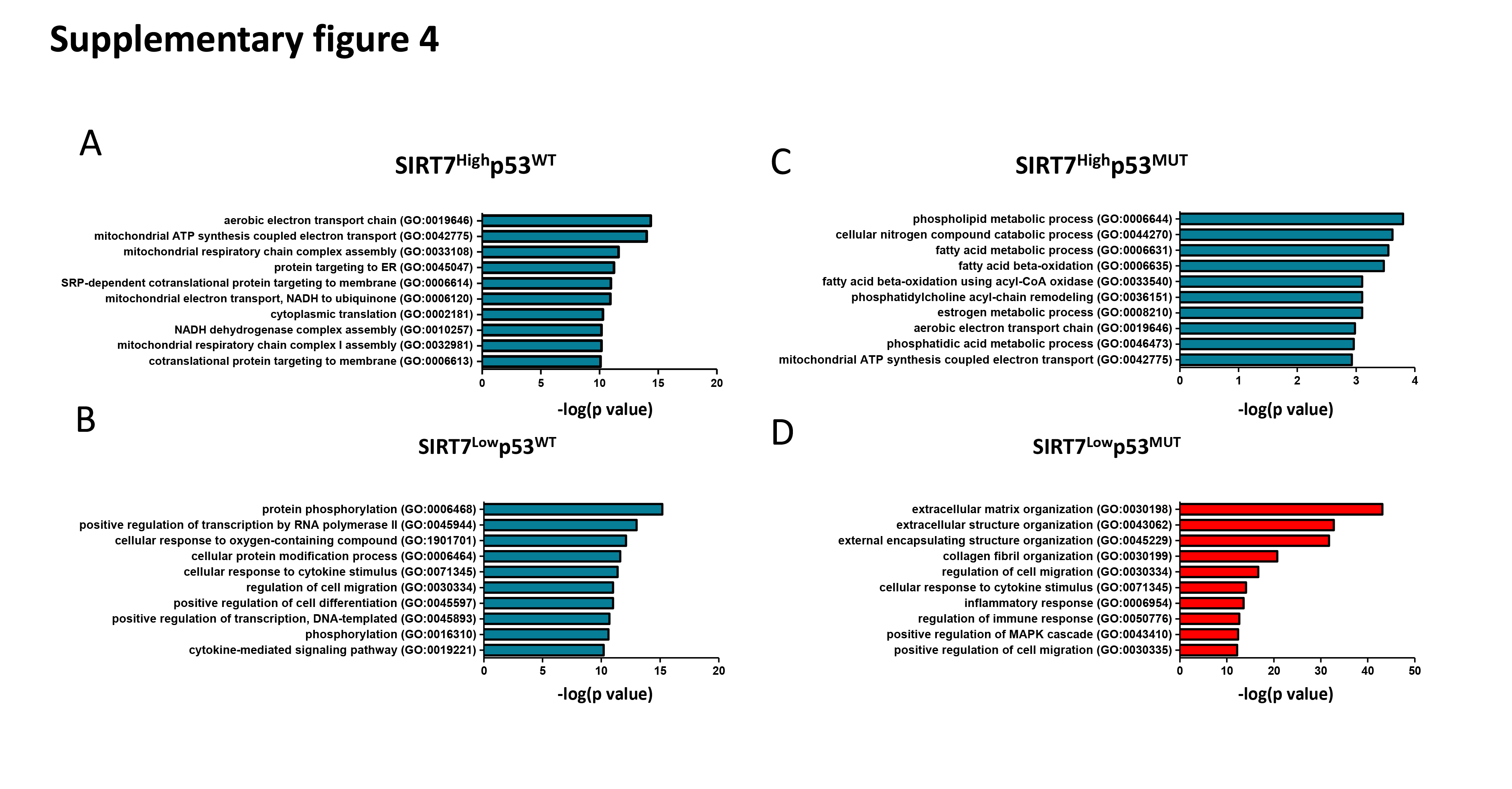

Supplement: Supplementary file 3 [file Image4.TIF]

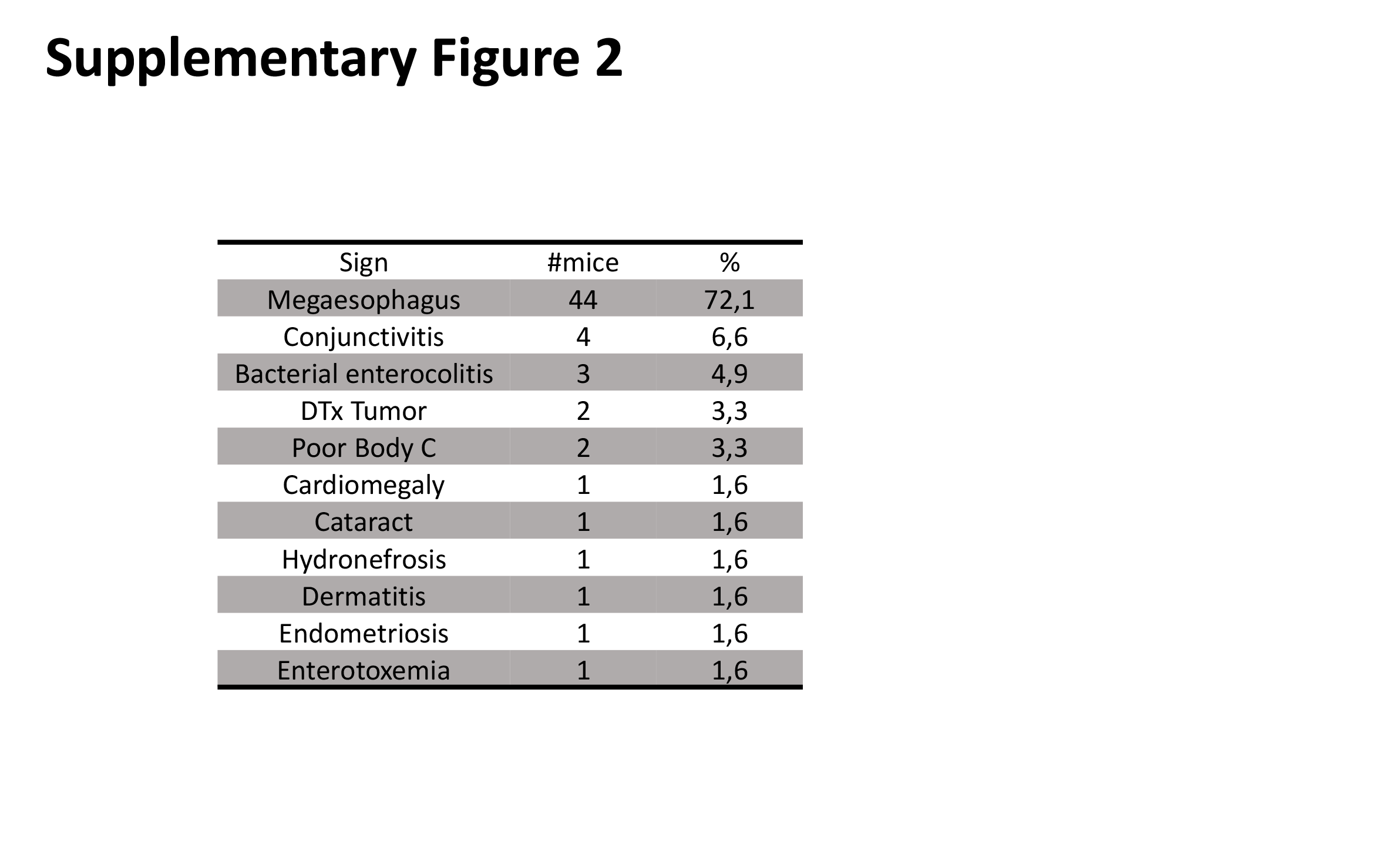

Supplement: Supplementary file 4 [file Image2.TIF]

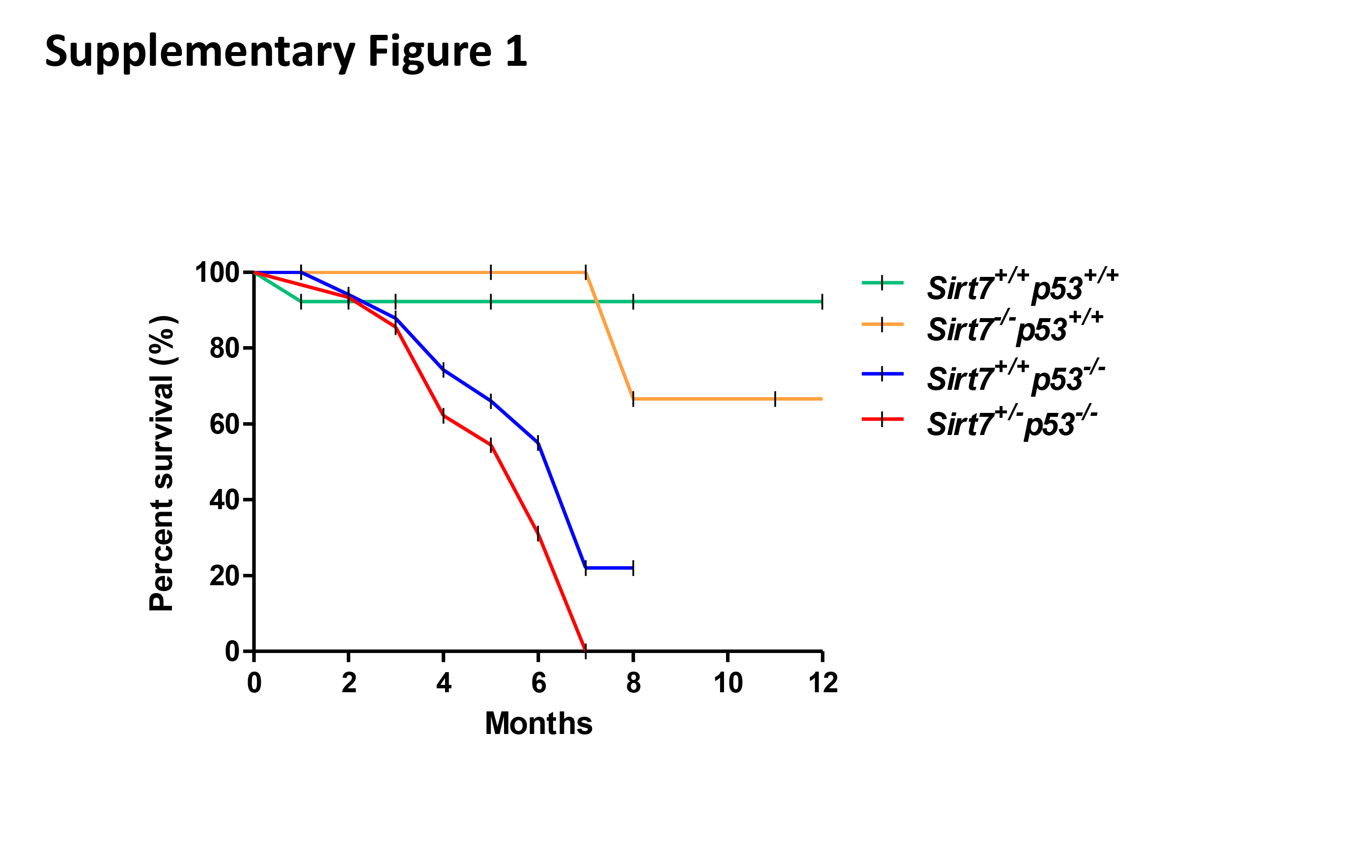

Supplement: Supplementary file 5 [file Image1.TIF]

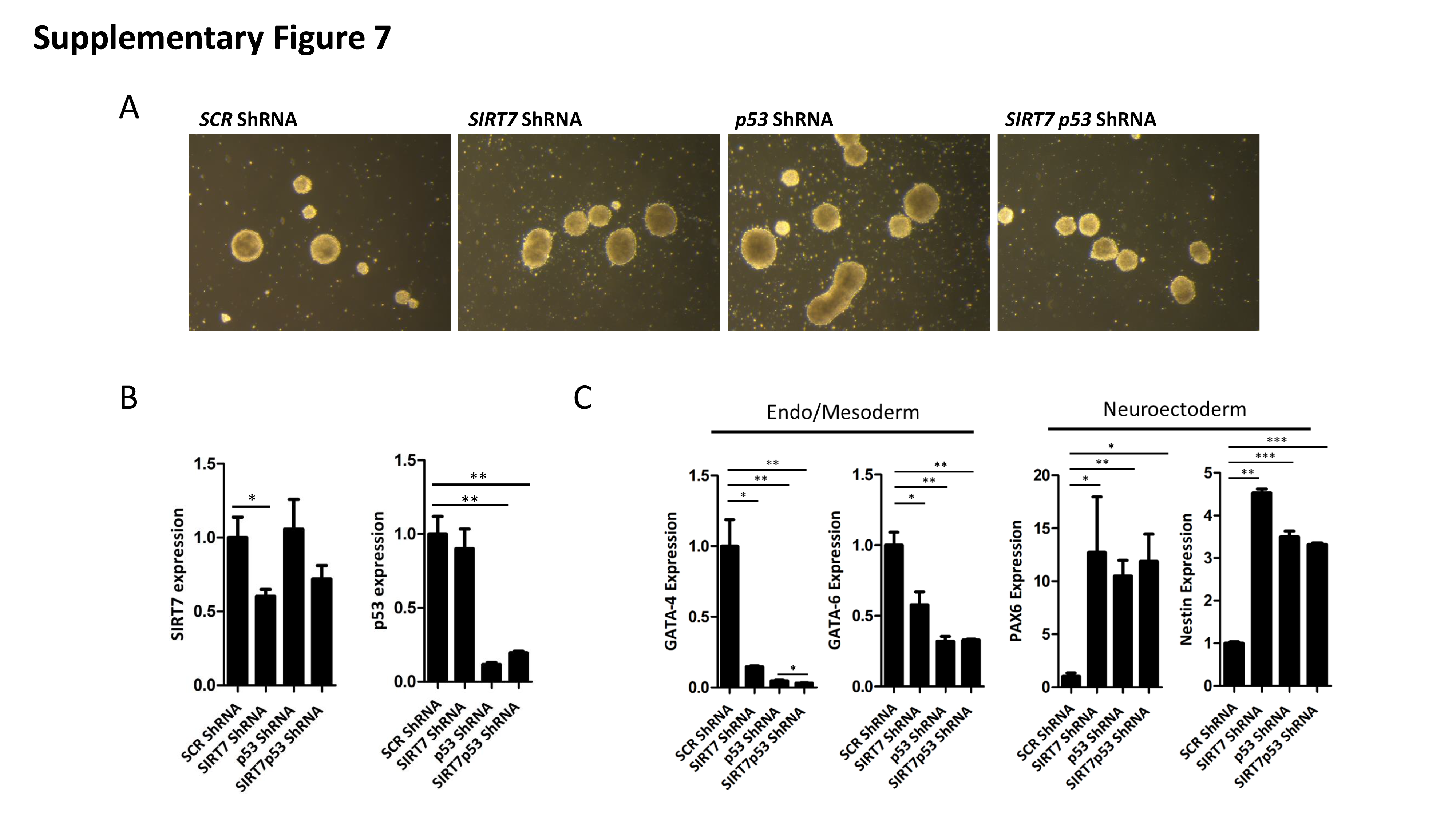

Supplement: Supplementary file 6 [file Image7.TIF]

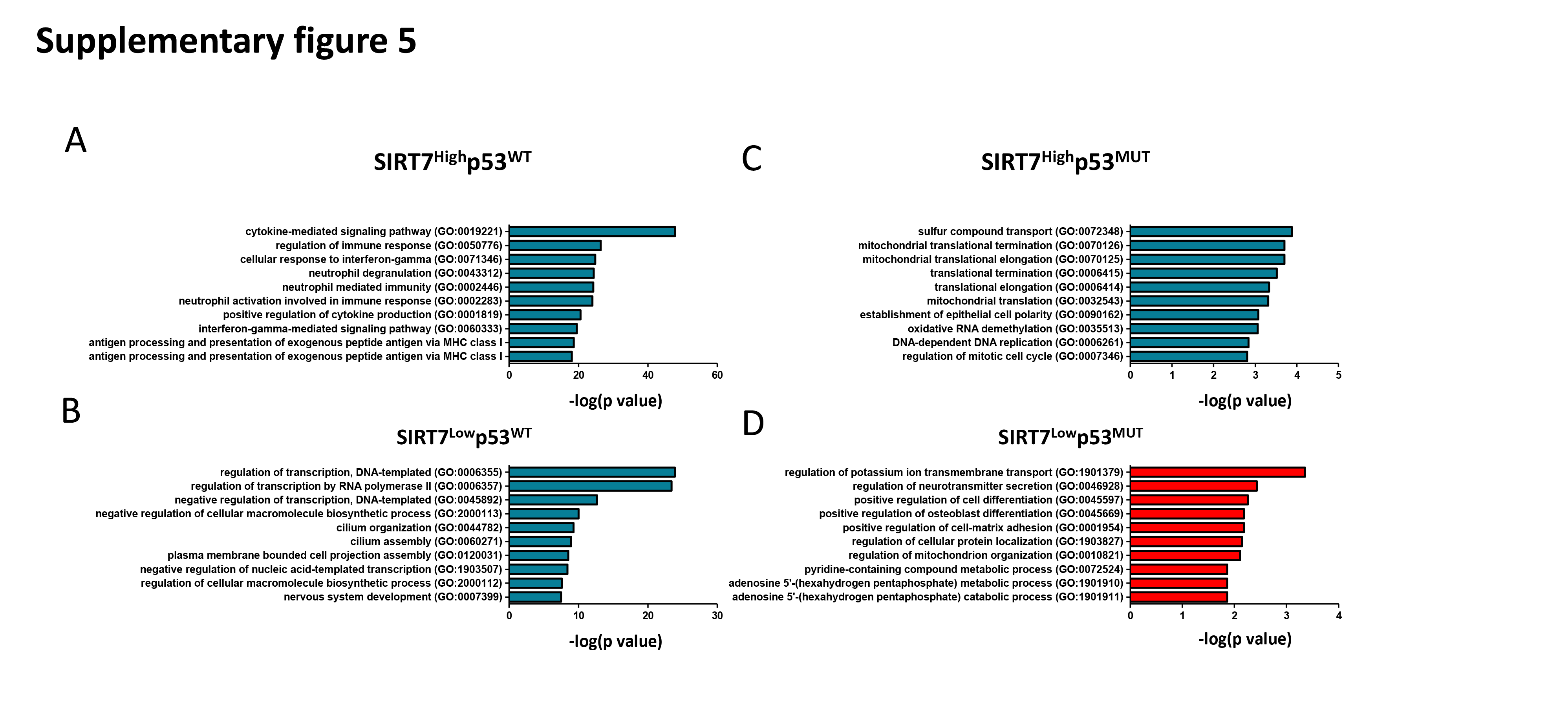

Supplement: Supplementary file 7 [file Image5.TIF]
